# Clinical correlates of grey matter pathology in multiple sclerosis

**DOI:** 10.1186/1471-2377-12-10

**Published:** 2012-03-07

**Authors:** Dana Horakova, Tomas Kalincik, Jana Blahova Dusankova, Ondrej Dolezal

**Affiliations:** 1 Department of Neurology and Center of Clinical Neuroscience, Charles University in Prague, 1st Faculty of Medicine and General University Hospital, Charles University, Prague, Czech Republic

## Abstract

Traditionally, multiple sclerosis has been viewed as a disease predominantly affecting white matter. However, this view has lately been subject to numerous changes, as new evidence of anatomical and histological changes as well as of molecular targets within the grey matter has arisen. This advance was driven mainly by novel imaging techniques, however, these have not yet been implemented in routine clinical practice. The changes in the grey matter are related to physical and cognitive disability seen in individuals with multiple sclerosis. Furthermore, damage to several grey matter structures can be associated with impairment of specific functions. Therefore, we conclude that grey matter damage - global and regional - has the potential to become a marker of disease activity, complementary to the currently used magnetic resonance markers (global brain atrophy and T2 hyperintense lesions). Furthermore, it may improve the prediction of the future disease course and response to therapy in individual patients and may also become a reliable additional surrogate marker of treatment effect.

## Review

Multiple sclerosis (MS) is known for the great variability of its clinical presentations, spanning the relapsing-remitting course with a subsequent secondary progressive phase, primary progressive course and relapsing-progressive course. The rate of disability accumulation varies from a lack of disease activity (benign MS) to rapidly progressing (malignant) MS [1] with a range of possible neurological manifestations. Therefore, the view of MS as a heterogeneous entity resulting from a number of inter-related etiopathogenetic cascades has been receiving increasing scientific attention [2-4]. The role of the immune system is likely to be pivotal in the disease pathogenesis, however, direct causality is yet to be established [5,6]. Surrogate markers such as magnetic resonance imaging (MRI), optic coherent tomography and susceptibility genes may elucidate the great clinical variability arising from the complex etiopathogenesis. On the diagnostic level, these might help to identify the specific subtypes of disease in individual patients, predict the future MS course, and develop individually tailored therapeutic regimens [7,8].

The currently available therapies, which are based mainly on their anti-inflammatory properties, are imperfect, with a number of patients showing only sub-optimal control over the MS activity [9]. It is therefore important that clinicians are able to predict the future response to treatment in individual patients early after disease onset in order to allow for the most appropriate treatment to be chosen [10]. Furthermore, the treatment, once administered, needs to be monitored to verify its efficacy. In both instances, surrogate markers may play significant roles [11,12]. Among different surrogate markers, MRI has been the only one used routinely in clinical practice. The traditional view of MS as a disease affecting predominantly white matter (WM) was driven by the higher sensitivity of the conventional MRI techniques to the WM changes [13-15]. However, these changes proved to be insufficient to explain the broad spectrum of neurological and psychological manifestations of MS satisfactorily [16-22]. Novel MRI techniques with improved sensitivity to grey matter (GM) changes [23-28] have shown that the GM damage is more prevalent than first estimated [29-34], that it may even precede development of the WM damage [35], and that it is significantly associated with physical and cognitive impairment [11,12,31,36-47]. The aim of this review is to summarise the current knowledge of the GM damage in MS and of its clinical implications.

### Assessing grey matter pathology

Both GM atrophy [11,34,38,41,42,44,48] and GM lesions [29-32,49-52] were demonstrated in cerebral cortex and deep GM structures using MRI supported by histological studies [32,53-56]. A body of work suggested that GM atrophy occurs early in relapsing-remitting as well as primary progressive MS [15,38,57-59]. Its progression was shown to be more prominent compared to WM atrophy, which is in contrast to some of the earlier works [12,33,34,44,60]. GM atrophy becomes more evident with the progression of MS [12,34,36] and in the chronic stages might even drive total brain atrophy [12]. Its relation to the WM changes, however, has not been sufficiently explained [52,61,62]. GM atrophy has been associated with several MHC II alleles [63], which are known genetic risk factors in MS [6,64]. This all implies that GM atrophy may play an important role in the pathogenesis of MS.

It is known that GM atrophy is not distributed homogeneously. Temporal and frontal cortex (including motor areas) can be affected predominantly, particularly early in the disease course [12,33,39,65-70]. The subcortical GM also shows marked atrophy, especially in the thalamus, basal ganglia (caudate and striatum) and the infratentorial structures [58,66,71,72]. As a result, cortico-subcortical connections might suffer significant damage [73].

According to the original pathological study of Brownell and Hughes, GM lesions comprise 26% of all lesions identified in the central nervous system (CNS) [29]. Cortical lesions occur early in clinically isolated syndrome (CIS) and relapsing-remitting MS, as well as in primary progressive MS (36%, 64% and 81% of patients, respectively) and increase in number and size with progression of the disease [30,31,74]. Cortical lesions are most common in the frontal and temporal cortex, predominantly affecting the motor (30-40%) and cingulate areas (10%) [75]. Among the subcortical GM, the structures most affected are the thalamus, basal ganglia, hypothalamus, hippocampus, cerebellum and spinal cord [76-80]. Compared to WM lesions, inflammation is less pronounced [51] and the blood-brain barrier is not disrupted in GM lesions [81]. Interestingly, T-cell mediated autoimmunity directed against contactin-2, which is present specifically within the GM, was identified as a factor contributing to the GM pathology in MS [3].

Sensitivity of the conventional MRI methods for GM lesions is low compared to WM lesions [32,82]. This improves with alternative techniques, such as double inversion recovery (DIR) [25,28,83] and its combination with phase-sensitive inversion recovery [27], T1-weighted gradient-recalled-echo [23] and higher field-strength MRI [24,26]. Another promising approach is the combination of the conventional MRI techniques with magnetisation transfer ratio [73,84]. Furthermore, diffusion tensor imaging has the potential to uncover progressive microstructural changes in normal-appearing GM [85]. Functional changes in MS can be examined using functional MRI to study re-organisation of the cortex, positron emission tomography to establish activation of microglia, or continuous arterial spin labelling to analyse brain perfusion [86-88]. Despite their promising results, the non-conventional MRI techniques have so far found only a limited use in routine clinical practice, partly due to their sparse availability and high technological and time requirements, and partly due to limited reproducibility of their outcomes [89].

### Clinical correlates of GM impairment

Abnormalities of GM are present early in CIS [90-95] and evolve with its progression to definite MS [11,96-98]. Numerous works have shown that the changes in GM are closely associated with both physical disability and cognitive impairment (see Table 1) [31,33,37,68,99-101].

**Table 1:** Selected works studying grey matter changes and their relations to physical and cognitive impairment in MS

Study	Patients	Follow-up (years)	MRI measures	Main outcomes
Dalton *et al*., 2004 [11]	58 CIS	3	GMF	Decrease in GMF was higher in patients who converted to CDMS (-3.3%) than in those who did not (-1.1%).

Fisher *et al*., 2008 [12]	7 CIS 36 RRMS 27 SPMS 17 HC	4	BPF, GMF, WMF	GMF decrease was more pronounced in patients compared to HC: CIS converting to RRMS, 3.4×; RRMS, 8.1×; RRMS converting to SPMS, 12.4×; SPMS, 14×. WMF decrease was 3× higher in all patient sub-groups than in HC.

Fisniku *et al*., 2008 [36]	29 CIS 33 RRMS 11 SPMS 25 HC	20/cross-sectional	GMF, GMV WMF	GMF but not WMF correlated with EDSS (r = 0.48) and MSFC sub-scores (r = 0.59).

Horakova *et al*., 2009 [102]	170 RRMS	5	GMV, PBVC	Decline in PBVC and GMV were the strongest MRI predictors of disability progression.

Calabrese *et al*., 2009 [30]	48 PPMS	2	GMF, cortical lesions (volume, count)	Baseline volume of cortical lesions correlated with EDSS (r = 0.48) and its change over 2 years (r = 0.38).

Calabrese *et al*., 2011 [96]	105 CIS 42 HC	4	Regional atrophy	Atrophy of the superior frontal gyrus, thalamus, and cerebellum predicted independently conversion from CIS to CDMS.

Roosendaal *et al*., 2011 [55]	95 CIS 657 RRMS 125 SPMS 50 PPMS	cross-sectional	GMV	GMV was lower in SPMS than RRMS, and was the strongest independent predictor of physical disability and cognitive impairment.

Amato *et al*., 2004 [40]	41 RRMS 16 HC	cross-sectional	GMV	Cortical atrophy was found in cognitively impaired but not in cognitively preserved patients, and was correlated with a poorer performance on tests of verbal memory, attention, and verbal fluency.

Amato *et al*., 2007 [101]	28 RR MS	2.5	GMV, NCV PBVC	Decrease in cortical volume was significantly higher in cognitively deteriorating than in stable or improving patients (-43 ml vs. -18 ml).

Tekok-Kilic *et al*., 2007 [68]	59 CDMS	cross-sectional	GMF	Frontal atrophy was associated with impaired memory (auditory/verbal, visual episodic and working).

Houtchens *et al*., 2007 [161]	62 RR MS 16 SP MS 1 PP MS 16 HC	cross-sectional	thalamic volume	Thalamic volume was 17% lower in the MS group than in HC, and was associated with impaired cognitive performance (r = 0.51-0.72) and physical disability (r = 0.32).

Calabrese *et al*., 2009 [37]	70 RR MS	cross-sectional	cortical lesions NCV	Higher number and volume of cortical lesions and lower volume of neocortical grey matter were seen in cognitively impaired vs. cognitively preserved patients.

BPF, brain parenchymal fraction; CDMS, clinically definite multiple sclerosis; CIS, clinically isolated syndrome; EDSS, Expanded Disability Status Scale; GMF, grey matter fraction; GMV, grey matter volume; HC, healthy controls; MS, multiple sclerosis; MSFC, Multiple Sclerosis Functional Composite; NCV, normalised cortical volume; PBVC, percentage brain volume change; RRMS, relapsing-remitting multiple sclerosis; SPMS, secondary progressive multiple sclerosis; WMF, white matter fraction

#### Physical disability

##### GM atrophy

It is known that GM atrophy is correlated with physical disability and its progression (r = 0.47 - 0.59) [12,36,39,102,103]. According to a number of studies, this relation is stronger than that of WM matter changes [33,57,67,99,100]. Fisniku and co-workers showed that GM atrophy, unlike WM atrophy, increases in patients with moderate disability [Expanded Disability Status Scale (EDSS) > 3] [36]. This view is further supported by the fact that the GM atrophy rate is accelerated upon conversion from CIS to the relapsing-remitting and secondary progressive stages (3.4× and 14× the normal rates, respectively), while WM atrophy remains stable throughout the MS course (3× the normal rate) [11,12]. The association of GM atrophy with disability becomes even stronger in primary progressive MS [33]. All this suggests that the GM changes could be more representative of the progressive damage to the CNS and the resulting physical disability than the WM damage. However, it is worth noting that also some contrasting results have been reported [15]. These opposing conclusions may relate to inequalities in studied cohorts, such as differences in disease stages or subtypes.

##### GM lesions

Apart from the GM atrophy, cortical and subcortical inflammatory (T2 hyperintense) GM lesions also contribute to the overall disability in MS [104,105]. They show mild correlation with EDSS and moderate correlation with its changes in time [31]. Similar to the atrophy, primary progressive MS shows more pronounced accumulation of the GM lesions, parallel with accumulation of the physical disability [30]. On the other hand, in a benign form of MS with only a modest disability after long disease duration, the GM lesions are sparse [106].

T2 hypointense lesions have also been reported in MS. They may represent iron deposits and foci of brain degeneration [107,108], predominantly located within the thalamus, striatum and rolandic cortex [107-109]. Similar to the T2 hyperintense lesions, T2 hypointense lesions are associated with physical disability [43,109-111] as well as cognitive impairment [112], and are predictive of future brain atrophy [108,113].

##### Regional GM changes

Among the regional GM changes, it is in particular the cortical atrophy which is thought to be associated with physical disability [13,15,33,100]. However, structural changes within the thalamus could also play role in the accumulation of disability [114]. It was suggested that MS-associated fatigue could be secondary to the regional atrophy of the fronto-parietal cortex, striatum and thalamus [115-118] as well as the higher overall GM lesion burden [69,119]. On the other hand, impaired gait may be associated with the damage to the dentate nucleus [43]. Another co-morbidity of MS - restless legs syndrome - is probably related to the changes in the cervical spinal cord [120], where demyelination of GM is more extensive than that of WM [77]. Apart from the routinely evaluated signs of the physical disability, the GM lesions are likely to contribute to the increased epileptic activity [121] which occurs in 2.9% of patients with MS (i.e. its prevalence is 3-6× higher compared to healthy population) [122-124]. Yet, it is not known whether the severity of physical impairment is proportional to the GM lesion volume or if it depends more on the topography of the focal GM damage.

##### GM reorganisation

Besides the limited regenerative capacity of the CNS [125], adaptation of neural networks represents important compensatory mechanism of the damaged CNS. Cortical reorganisation, as shown by a number of studies with functional MRI, occurs early in MS, but its extent varies greatly among patients. It can be visualised as a non-normal cortical activation pattern, elicited by standardised motor and cognitive tasks [126-133]. For instance, during motor processing, recruitment of higher (supplementary) areas may be seen even with simple movements in MS patients but not in healthy subjects [129,130,134]. Similar functional reorganisation takes place in the cervical spinal cord [135]. This can be interpreted as compensation for damage inflicted by the demyelination and neuronal loss. It is possible that more extensive (or efficient) compensation and axonal regeneration contribute to a less severe course of MS and slower accumulation of the CNS structural damage [106,134].

##### Evaluation of disability

Research of functional outcomes of the structural changes in MS depends on the ability of clinicians to quantify physical and cognitive impairment in MS patients. Two scales, EDSS and Multiple Sclerosis Functional Composite (MSFC), have been used most commonly to evaluate the physical impairment in clinical practice and in research. Both of these scales quantify the extent of disability only imperfectly [136]. For EDSS, this is attributed to suboptimal inter-rater reproducibility, lack of weighted functional sub-scores and omission of psychological assessment [137], while for MSFC, this is due to practice effects, variations in reference populations, omission of visual assessment and lack of accepted definition of a clinically meaningful change [138]. EDSS mainly evaluates the physical component of the impairment, with the emphasis on ambulation, assessing the cognitive impairment only marginally. On the other hand, MSFC is a more complex scale with objective evaluation of ambulation (timed 25-foot walk test), fine motor skills (9-hole peg test) and cognition (3-second Paced Auditory Serial Addition Test). It was suggested that MSFC may better correlate with GM atrophy than EDSS [12,36]. Furthermore, it is possible that EDSS is more sensitive to disability progression in patients with mild physical disability, while being less sensitive to the progression in patients with more severe disability [139]. This raises concerns about the value of EDSS in secondary progressive MS. In any case, instruments assessing the physical disability reliably at all stages and in all courses of MS are critical for accurate evaluation of the descriptive and prognostic value of the GM changes.

#### Cognitive impairment

Cognitive impairment is highly prevalent in MS, affecting 40-65% of patients with all disease courses and in all its clinical stages [140]. Although the character and severity of the cognitive impairment vary widely among the patients, information processing speed, attention, recent and long-term memory, executive functions and visuospatial abilities seem to be the most affected domains, whereas general intelligence, language and certain aspects of memory (short-term capacity and implicit memory) are spared, and overt dementia is rare in MS [141-143]. In addition, in patients with disease onset before the age of 18, impairment of expressive language and visuomotor integration were described [144]. This suggests that even in young patients the damage to the CNS may exceed its plasticity. Overall, the extreme variability of the cognitive impairment may depend on several factors, such as patient age, gender, age at disease onset, level of education and cognitive reserve [145,146].

##### GM vs. WM changes

Even though significant correlations between the amount and the regions of the WM atrophy vs. the degree and pattern of cognitive impairment were shown [147], studies failed to explain the full array of cognitive impairment by the WM damage only [148]. A range of specific cognitive deficits, such as memory impairment, low information processing speed and attention deficits, could be better explained by the cortical GM lesions rather than the subcortical WM lesions [148]. Changes in the GM might therefore add to our understanding of the causality of the cognitive impairment in MS. For example, more widespread atrophy and hypometabolism of GM can be found in the cognitively impaired patients than in those cognitively intact [149,150]. Moreover, it is of interest that the cognitive impairment is more prominent at the time of conversion from the relapsing-remitting to the secondary progressive course [151,152], which is also marked by accelerated degeneration of the cerebral GM [12]. In fact, a number of works provided evidence of a strong association between GM impairment (lesions and atrophy) and global or selective cognitive disability in MS [40,68,101,142,149,153], which may imply a causative relation [148].

##### Regional GM changes

A pattern of widespread cortical thinning was found in cognitively impaired patients with relapsing-remitting MS [149,154]. Even a cortical variant of MS was described in those with the cognitive impairment among the initial manifestations of MS [155,156]. It was shown that neocortical atrophy is related to the impairment of verbal memory [40,65,68,153], visual episodic and working memory [68], verbal fluency [40,101], attention/concentration [40] and processing speed [65,70,157]. It may also be responsible for subtle personality changes observed in MS patients, such as disinhibition and euphoria [153,158]. More specifically, atrophy of the prefrontal, precentral and superior parietal cortex is related to the decreased processing speed and impaired calculation abilities [70]. Left frontal atrophy occurs in patients with impaired auditory/verbal memory, while right frontal atrophy is related to impaired visual episodic and working memory [68]. Atrophy of the mesial temporal cortex is associated with decreased processing speed and impaired episodic and verbal memory [159,160]. Atrophy of the subcortical GM structures can be evaluated either directly or indirectly - using enlargement of the third ventricle as a marker [68,154]. Of the subcortical GM, the most relevant are the atrophy, structural changes and altered metabolism of the thalamus, which are linked with deterioration in multiple cognitive domains [114,144,150,154,157,161,162].

Compared to GM atrophy, there is considerably less evidence to support contribution of demyelinating lesions of GM to cognitive impairment. The volume of the cortical lesions shows only a modest association with cognitive impairment, while an increase in the lesion volume seems to be moderately associated with cognitive deterioration [31,37,163,164]. More specifically, lesions in the medial frontal and temporal cortex seem to correlate with impaired memory [164].

Overall, it can be speculated that the cognitive decline observed in MS patients results from focal inflammatory lesions and widespread GM loss. Despite the fact that the neuropsychological profiles of MS patients cannot be defined as either purely "cortical" or "subcortical" [165], it is likely that it is the impairment of the cortical GM which determines the level and character of cognitive dysfunction.

### GM as a surrogate marker

Objective indicators of MS activity as well as predictors of future disease course and treatment efficacy applicable in individual patients are crucial for making appropriate therapeutic decisions in routine clinical practice. A number of works have addressed these issues, and several markers, both clinical and paraclinical, have been suggested [7,21,166-169]. Yet, accuracy of the MRI markers, particularly when used in individual patients, is only limited [16,170,171].

#### Marker of MS activity

According to the existing evidence, changes in GM might represent a reliable marker of disease activity and of CNS damage. The relatively less pronounced inflammation within GM is likely to result in lesser fluctuations of its changes triggered by the relapsing inflammatory activity [51]. Moreover, focal oedema and treatment-associated pseudoatrophy, which may mask the changes reflecting the activity of MS, are known to be less evident in GM [172,173]. Therefore GM lesions and atrophy, rather than WM changes, might better reflect long-term changes which drive the accumulation of disability [174].

In fact, assessment of GM lesions improves the specificity and accuracy of MRI diagnostic criteria [175]. At the same time, GM atrophy correlates closely with the progression of CIS to clinically definite MS [11,12,39,176]. Furthermore, both GM lesions and GM atrophy can be used to predict this conversion [96,175]. Long-term accumulation of disability is also predicted by the diffuse changes in the GM [36,177]. It can be speculated that an even better prognostic value may be achieved with assessment of regional GM atrophy.

#### Monitoring of treatment efficacy

For the reasons discussed above, the impairment of GM has the potential to become an important marker of the efficacy of immunomodulatory remedies [21]. On the other hand, the less inflammatory nature of GM damage [51,178,179] and better preservation of the blood-brain barrier within altered GM [180] may diminish the response of GM to immunomodulatory therapy. Calabrese and co-workers demonstrated a decrease in accumulation of GM lesions and cortical atrophy in patients treated with disease modifying drugs, and reported a more pronounced effect of subcutaneous interferon β compared to intramuscular interferon β and glatiramer acetate [181]. Zivadinov and co-authors observed ameliorated progression of GM atrophy in patients treated with interferon β [182]. In contrast, Benfeldt and co-workers reported more pronounced atrophy in the fronto-temporal, cingulate and cerebellar cortex in patients treated with interferon β. It is therefore apparent that more work evaluating the effect of immunomodulation on the changes in GM is required.

## Conclusions

The growing body of evidence supports the view of MS as a disease not only of WM but also of GM. The mechanisms responsible for the inter-individual variation in the extent of GM and WM pathology are largely unknown, and their identification will significantly contribute to the understanding of the MS etiopathogenesis. On the diagnostic level, GM atrophy and lesions provide information complementary to the conventional MRI variables and further improve correlation between the radiological and clinical variables [118,183]. Thus, GM pathology may not only serve as a new marker for the existing immunomodulatory therapies but may also provide a potential target for novel therapies.

## Abbreviations

CIS: clinically isolated syndrome; CNS: central nervous system; EDSS: Expanded Disability Status Scale; GM: grey matter; MRI: magnetic resonance imaging; MS: multiple sclerosis; MSFC: Multiple Sclerosis Functional Composite; WM: white matter

## Competing interests

Dana Horakova has received speaker honoraria and consultant fees from Biogen Idec, Merck-Serono, Bayer Shering and Teva, as well as support for research activities from Biogen Idec.

Tomas Kalincik has received compensation for travel and honoraria from Biogen Idec, Sanofi Aventis, Teva and Merck-Serono.

Jana Blahova Dusankova has received speaker honoraria and compensation for travel from Biogen Idec, Bayer Shering, Merck-Serono and Novartis.

Ondrej Dolezal has received speaker honoraria and compensation for travel from Biogen Idec, Merck-Serono and Novartis.

The authors have received financial support from the Czech Ministry of Health [MSM 0021620849].

## Authors' contributions

DH prepared the *Introduction *and *Physical disability *sections and Table 1, and reviewed the manuscript. TK prepared the *Physical disability *section and Table 1, and edited and reviewed the manuscript. JBD prepared the *Cognitive impairment *section and reviewed the manuscript. OD prepared the *Assessing grey matter *pathology section and reviewed the manuscript. All authors read and approved the final manuscript.

## Pre-publication history

The pre-publication history for this paper can be accessed here:

http://www.biomedcentral.com/1471-2377/12/10/prepub
